# *Plasmodium vivax* Seroprevalence in Bred Cynomolgus Monkeys, China

**DOI:** 10.3201/eid1705.101846

**Published:** 2011-05

**Authors:** Hai-Long Li, Zhen-Yong Liu, Juan Li, Lin Ai, Dong-Hui Zhou, Zi-Guo Yuan, Rui-Qing Lin, Ya-Biao Weng, Xing-Quan Zhu

**Affiliations:** Author affiliations: State Key Laboratory of Veterinary Etiological Biology, Lanzhou Veterinary Research Institute, CAAS, Lanzhou, China (H.-L. Li, Z.-Y. Liu, J. Li, L. Ai, X.-Q. Zhu);; South China Agricultural University, Guangzhou, China (H.-L. Li, J. Li, L. Ai, D.-H. Zhou, Z.-G. Yuan, R.-Q. Lin, Y.-B. Weng)

**Keywords:** Plasmodium vivax, malaria, parasites, seroprevalence, cynomolgus monkeys, Macaca fascicularis, China, letter

**To the Editor:** Malaria caused by *Plasmodium* spp. is one of the most prevalent parasitic diseases in the world, especially in tropical countries. *P.* *vivax* represents the second most prevalent of malaria species. Therefore, as measures to control the high death rates in humans caused by *P. falciparum* become more effective, *P. vivax* is likely to become the primary malaria threat (*1*). In *P. vivax* infection, the main clinical signs and symptoms are fever, chills, nausea and vomiting, generalized body pain and fatigue, toxic shock and pulmonary edema, retinal hemorrhage, renal failure, uremic encephalopathy, and thrombocytopenia (*2*–*6*).

Because of their phylogenetic proximity to humans, nonhuman primates have been used extensively as animal models of human diseases (*7*). Thousands of workers come in contact with monkeys, but little information about the prevalence of *P. vivax* in bred cynomolgus monkeys (*Macaca fascicularis*) is available worldwide. The objective of our investigation was to examine whether *P. vivax* seroprevalence is present in bred cynomolgus monkeys in the People’s Republic of China.

A total of 328 blood samples were collected by venous puncture during June 2008–September 2009. Of these, 224 blood samples were from 4 nonhuman primate centers in Guangxi Zhuang Nationality Autonomous Region and 108 blood samples were from 2 nonhuman primate centers in Guangdong Province. All of the cynomolgus monkeys were caged. Each cage has 2 rooms, 1 indoors and 1 outdoors. The monkeys spend ≈10 hours in the outdoor room each day during the daytime. The age and sex of monkeys are listed in the Table. Serum samples were separated and stored at –20°C before testing.

Serum samples were tested for *P. vivax* antibodies by using a commercially available ELISA kit (Tiancheng Yiliu Co., Ltd, Shanghai, China), according to the manufacturer’s instructions. This kit uses biotinylated anti–*P. vivax* as a coating antigen and is specifically for monkeys. Positive and negative control serum samples were provided in the kit and included in each test. Those samples with doubtful results were retested.

Differences in the seroprevalence of *P. vivax* in bred cynomolgus monkeys according to sex and area were analyzed by using the χ^2^ test in SPSS 13.0 standard version for Windows (SPSS Inc., Chicago, IL, USA). The differences were considered to be statistically significant when the p value obtained was <0.05.

The total prevalence of anti–*P. vivax* antibodies in bred cynomolgus monkeys in southern China was 3.4% (11/328), which was lower than the prevalence of anti–*P. vivax* antibodies in captured monkeys (*Alouatta seniculus, Saguinus midas,* and *Pithecia pithecia*) in French Guiana (*8*). The prevalence in female monkeys (3.1%, 5/161) was slightly lower than that in male monkeys (3.6%, 6/167); the seroprevalence of 3.6% (8/224) in Guangxi Zhuang Nationality Autonomous Region was slightly higher than that in Guangdong Province (2.9%, 3/104) (Table), but these differences were not significant (p*>*0.05). A total of 52 monkey serum samples from nonhuman primate center E in Guangdong Province were found seronegative for *P. vivax* antibodies (Table). The difference in prevalence of *P. vivax* antibodies in different nonhuman primate centers may be related to differences in ecologic and geographic conditions, climate conditions, as well as in the management practices. All of the nonhuman primate centers are surrounded by hills or paddy fields, and the environment is favorable for *Anopheles* mosquitoes. The mosquito control measures, including the use of antimosquito insecticides and good drainage facilities for preventing water collection on the ground, were better executed at nonhuman primate center E than at the other nonhuman primate centers.

**Table:** Prevalence of *Plasmodium vivax* antibodies in serum samples, from bred cynomolgus monkeys in southern China, determined by ELISA, 2008–2009*

Province	Primate center	Female				Male				Total	
		Age, y	No. positive/ no. examined	Prevalence, %		Age, y	No. positive/ no. examined	Prevalence, %		No. positive/ no. examined	Prevalence, %
GX	A	3.0–5.0	0/20	0		3.0–6.5	1/30	3.3		1/50	2
B	3.0–5.0	0/29	0		3.0–5.5	1/33	3.0		1/62	1.6
C	3.0–5.0	1/27	3.7		3.0–5.5	2/33	6.1		3/60	5
D	2.0–5.0	2/31	6.5		3.0–5.0	1/21	4.8		3/52	5.8
Subtotal	2.0–5.0	3/107	2.8		3.0–6.5	5/117	4.3		8/224	3.6
GD	E	2.5–3.0	0/14	0		2.5–3.0	0/13	0		0/27	0
	E	7.0–8.0	0/12	0		8.0–9.0	0/13	0		0/25	0
	F	2.5–3.0	2/28	7.1		2.5–6.0	1/24	4.2		3/52	5.8
	Subtotal	2.5–8.0	2/54	3.7		2.5–9.0	1/50	2		3/104	2.9
Total		–	5/161	3.1		–	6/167	3.6		11/328	3.4

*GX, Guangxi Zhuang Nationality Autonomous Region; GD, Guangdong Province; –, not applicable.

Our survey showed *P. vivax* seropositivity in 5 of the 6 nonhuman primate centers in southern China, which is a potential health problem for bred cynomolgus monkeys. This finding also indicates the risk for infection with *P. vivax* for the employees of these nonhuman primate centers. Therefore, studies are warranted that assess the seroprevalence of *P. vivax* infection in persons who work in these nonhuman primate centers, as well as the seroprevalence of *P. vivax* infection in wild monkeys.
